# Actions Are Needed to Deal with the High Uncertainties in Tire Wear Particle Analyses

**DOI:** 10.1021/acs.est.3c02393

**Published:** 2023-06-02

**Authors:** Elisabeth Rødland, Yan Lin

**Affiliations:** The Norwegian Institute for Water Research, Økernveien 94, 0579 Oslo, Norway

**Keywords:** tire road wear particles, rubbers, uncertainty analysis

Better uncertainty estimation for tire wear particles in the environment and establishment of an open-source tire database would increase the strength of the data collected and improve the efficiency of mitigation efforts.

Tire road wear particles (TRWPs) containing synthetic rubbers are estimated to be one of the largest sources of microplastics to the environment.^1^ Different from other major types of plastics (PE, PP, and PS), for which many alternative materials are being developed and tested, there seems to be no quick alternatives to synthetic rubbers. With the development of road traffic, the level of emission of microplastics from TRWPs is likely to remain high for some time. These particles are created through the friction between tires and the road surface, creating abrasion particles from both sources, and they exhibit large morphology, size, density, and chemical property ranges.^2^ The question of whether tire wear rubbers truly belong within the scope of microplastics is debated, having chemistry, morphology, density, shape, and size distribution very different from those of the more commonly known plastic particles. However, it is unambiguous that TRWPs are potentially harmful to the environment, as they are linked to toxic effects in both aquatic and terrestrial organisms, especially due to certain tire additives such as 6-PPD-quinone.^3^ TRWPs have been found to accumulate in soils and sediments from road runoff and air deposits.^2^

Most of the current studies calculate the TRWP levels indirectly on the basis of the analysis of certain chemical markers in TRWPs, such as rubbers (SBR, BR, and NR) or zinc, benzothiazoles, and 6-PPD-q, which are typical additives in the synthetic rubbers, and the sample numbers are usually quite small. The issue with using an assumed marker level for all tires has previously been identified by studies concerning variations in the rubber content of different types of tires.^4^ By assuming a fixed level of, for example, synthetic rubber in all tires, there is a fair chance of either over- or underestimating the true value in the sample. For example, Rauert et al.^4^ demonstrated that the assumed 50% synthetic rubber concentration in tires would underestimate the TRWP levels by a factor of at least 5 compared to the true rubber concentration measured in various tires.

**Figure 1:**
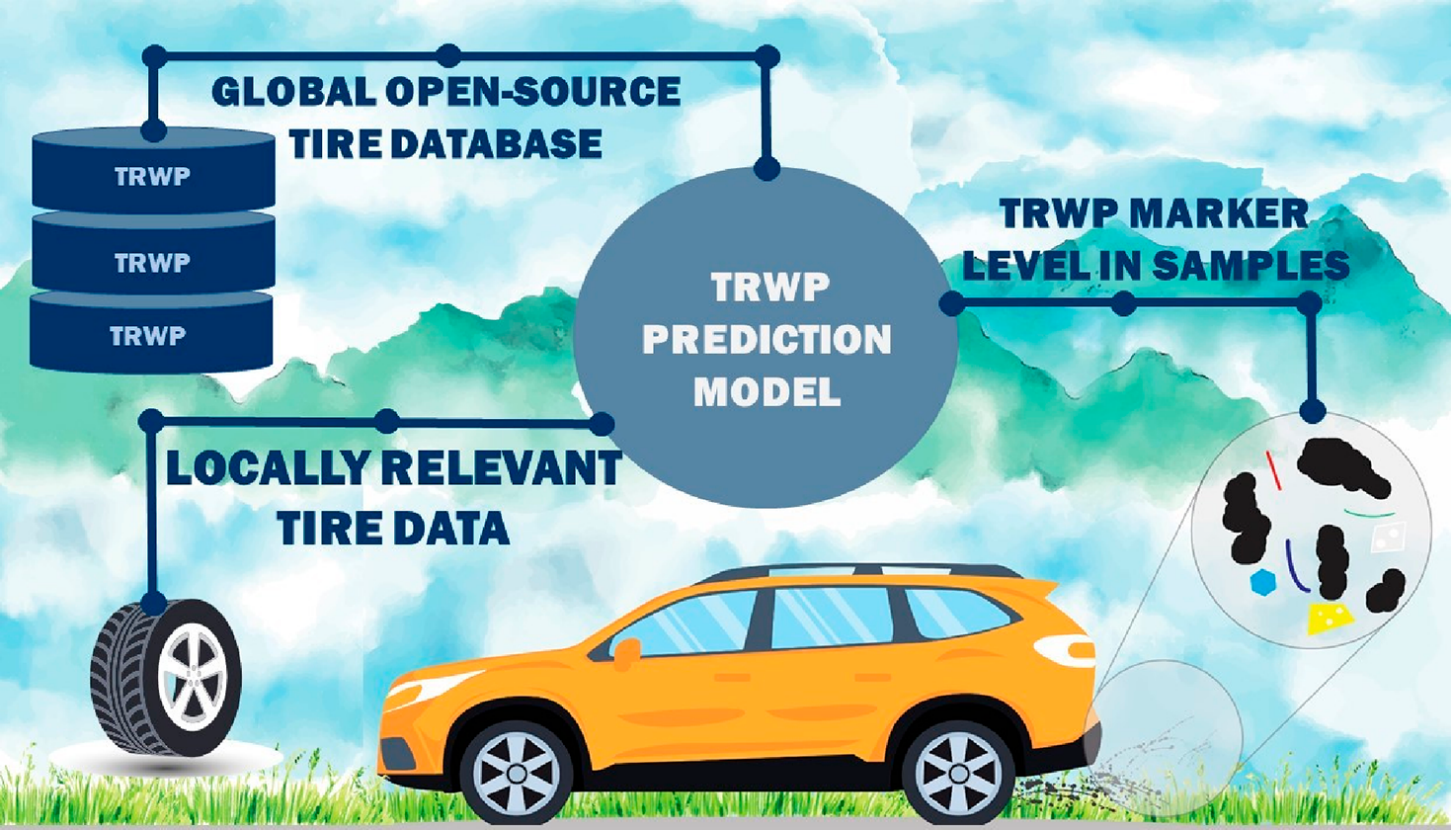
Schematic illustration of the prediction modeling of TRWPs, using a global open-source tire database with various TRWP markers, local data on the use of different types of tires, and the measured levels of TRWP markers in a sample. Graphics created with vectors from Macrovector, Freepik.

As the environmental concentrations are used to understand the processes related to the release of TRWP, such as their relationship with traffic density or traffic speed, which again is used to develop policies for mitigation efforts and evaluating environmental risks, this level of uncertainty is unacceptable. However, by using prediction modeling (Figure 1), it is possible to determine not only the average concentration of TRWP but also the source of uncertainties in each sample, as for example is possible by Monte Carlo analysis of TRWPs.^5^

In mass-based analysis of TRWPs, the results are indirectly based on various markers in tires. It is therefore important to improve the estimation of TRWP by providing the sources of uncertainties, and the results could be adjusted to local scale by using relevant tires for the area or the season. Another challenge for the current TRWP analytical methods is that there is not a good inventory of constituents in car tires on the market. The current European chemical legislation REACH (The Regulation on the registration, evaluation, authorization and restriction of chemicals) includes only a small fraction of the compounds in tires, and none of the compounds currently used as markers for quantification of TRWP are included in the list.^6^

Due to the lack of legislation governing the compounds in tires, information regarding compounds and levels associated with different types of tires remains unavailable to environmental authorities, researchers, and the public. We therefore propose that the research community, the tire industry, and the public sectors collaborate to create a global open-source tire database in which information about levels of markers used for quantification, such as the synthetic rubbers, is shared. The database should include different search options, in which different tire types based on season, vehicle type, country or region, and potentially age of the tires, should be important search options included among others. Data collected from various research studies, including exposure tests and aging experiments, and data about the tire constituents and production volume from the tire industry would be important to include. Such a database could reduce the uncertainties of TRWP release estimates considerably, and discussions on what information should be included should take place among the research community, the tire industry, and the general public as soon as possible. There are already various platforms established for such discussions, such as the European TRWP Platform and the Tire Industry Project, yet an open debate on how to create a global open-source database is still in its infancy. We acknowledge that there are potential hurdles to overcome to make such an open-source tire database, including how to keep an open and transparent process as well as ownership and funding of such a database. We also acknowledge that there is a potential conflict for the tire industry when it comes to sharing information that could be confidential. To overcome this, information about the brand of tires or other types of information that is proprietary should not be disclosed in such a database. The registration and listing of this information should happen at the chemical or environmental authorities, such as ECHA or other relevant authorities. Inquiries by publicly funded research projects under certain conditions should be granted access to this information. It is important to look at TRWPs from multiple sides to be able to tackle the problem. The industry, the researchers, and the public sector all have an interest in reducing the impact on the environment as much as possible, and in our opinion, the only way forward is together and by improving both the data available and how we interpret these data through uncertainty analysis.
